# Full-Thickness Macular Hole: An Exceptional Case Presentation in Behçet’s Disease

**DOI:** 10.7759/cureus.57599

**Published:** 2024-04-04

**Authors:** Asna Tahir, Hamza Rehman, Salman Tauqeer, Muhammad Usman Khan, Sadia Sethi

**Affiliations:** 1 Ophthalmology, Khyber Teaching Hospital, Medical Teaching Institution, Peshawar, PAK; 2 Ophthalmology, Hayatabad Medical Complex, Peshawar, PAK; 3 Internal Medicine, Cumberland Infirmary, Carlisle, GBR

**Keywords:** behcet's disease, painless vision loss, recurrent aphthous ulcers, behcet’s syndrome, full-thickness macular hole, pan-uveitis, chronic uveitis, auto immune, rare clinical presentation

## Abstract

Behçet’s disease (BD) is a vascular disorder affecting a variety of organ systems. It is an auto-immune disease with inflammatory vasculitis that is systemic in nature, the exact etiology of which is unknown. Obliterative vasculitis, recurrent aphthous ulcers, mucocutaneous manifestations, recurrent genital ulcerations, and intraocular inflammation, especially chronic relapsing uveitis, are the characteristic features of BD.

The case report presents a unique manifestation of BD in a 20-year-old Pakistani male who presents with a one-year-old history of viral encephalitis, after which he developed a blurring of vision. On examination, he had recurrent aphthous ulcers, recurrent ulcerations of genitalia, and a history of lesions of the skin. After making the patient undergo a cascade of investigations for evaluating and assessing the various signs and symptoms, a diagnosis of BD with bilateral panuveitis and a full-thickness macular hole (FTMH) in the right eye was established. Immuno-suppressants, steroids, and azathioprine were used as treatment options, following which the state of remission was attained.

## Introduction

Behçet’s disease (BD) is an autoimmune disorder that involves multiple systems of the body, following a course involving relapses and remittances. Mucocutaneous, ocular, and gastrointestinal (GI) symptoms are its frequent manifestations. The medical literature shows that this disease can manifest as a wide array of signs and symptoms involving several bodily organs and systems, which may include skin, genital, ocular, and neurological, in addition to other systems [1].

This case study brings into light a particular case of ocular involvement, showing right retinal detachment (RD) with a full-thickness macular hole (FTMH), which is a rare manifestation of BD. Uveitis in BD is typically non-granulomatous and can present as anterior uveitis, intermediate uveitis, posterior uveitis, or panuveitis. Panuveitis is the most frequent type (60%) in both sexes [2].

A small fraction of patients suffering from BD have reported a rare complication depicting the involvement of the macula, observed in 3.4% of cases [3]. In posterior uveitis, the most common complication usually observed is macular edema, which can then develop into cystoid degeneration and sometimes can lead to the formation of a macular hole (MH). The lesions involving the macula, particularly its ischemia, are irreversible in most cases, thus leading to an uncertain prognosis [4]. A typical presentation of BD involves a recurrence of oral as well as genital ulcerations along with uveitis or other such ocular symptoms [5].

Prevalence of BD has been reported to be 42/10,000 population of Turkey [6] and 80/100,000 population of Iran [7]. The presentation of such cases in this part of the world, that is, Southeast Asia, is rare and thus unique in medical literature.

Herein is a case presentation of a young male who presented with the complaint of recurrence of oral as well as genital ulcerations, along with decreased vision in the left eye since the last 15 days as the presentation of BD.

## Case presentation

**Figure 1:**
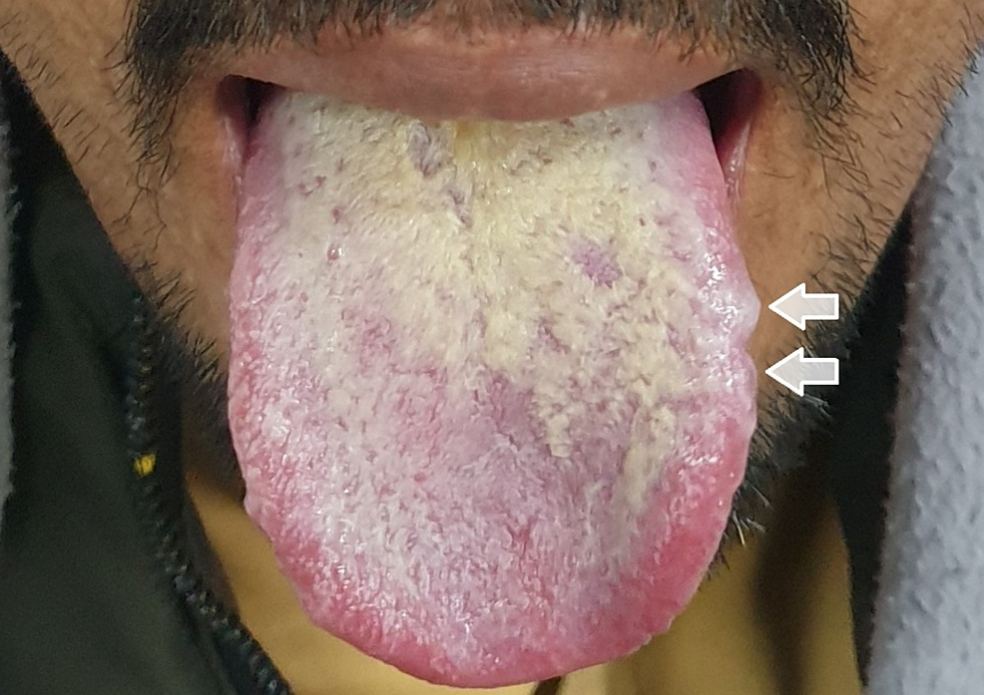
Aphthous ulcers on the left side of the tongue (white arrows).

A Pakistani male patient, aged 20, presented to the outpatient department (OPD) of our tertiary-care hospital in Peshawar with a complaint of gradual and painless visual impairment affecting his left eye for 15 days. On clinical examination, he appeared pale with aphthous ulcers on the tongue (Figure 1). The patient also gave a history of viral encephalitis one year ago.

**Figure 2:**
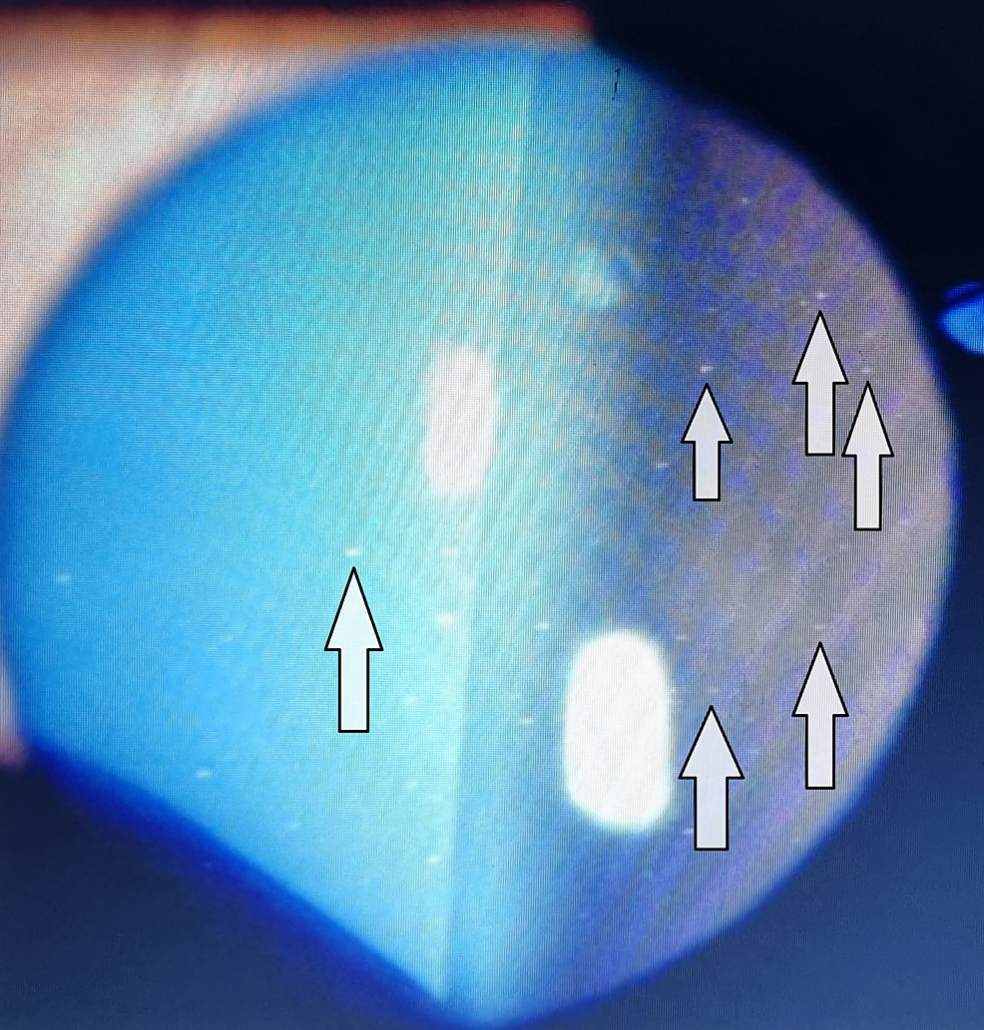
Anterior chamber photo showing +3 cells (white arrows) indicating uveitis.

**Figure 3:**
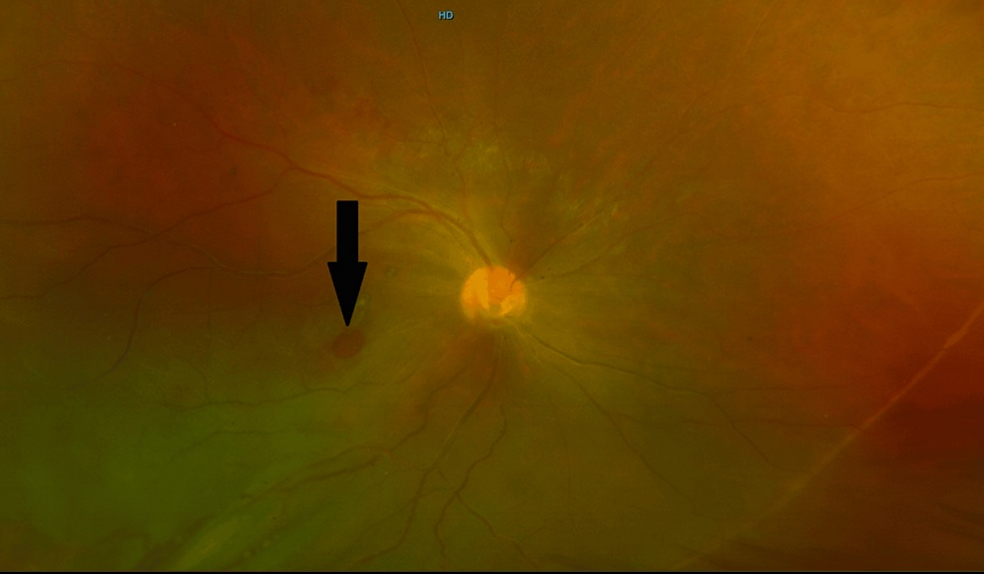
A color photograph of fundus of the right eye showing FTMH (black arrow). Moreover, the right retina was ischemic with vasculitis and proliferation. FTMH, full-thickness macular hole

The ocular examination of the patient revealed his visual acuity (VA) to be counting fingers in the right eye, while the VA of the left eye was hand movement. In the right eye, +3 cells were seen in the anterior chamber, which indicates anterior chamber reaction signifying uveitis (Figure 2). Fundus examination revealed bilateral panuveitis and bilateral vitritis (more in the left eye than the right), and in the right eye, the retina was detached. FTMH was also seen in the right eye (Figure 3).

**Figure 4:**
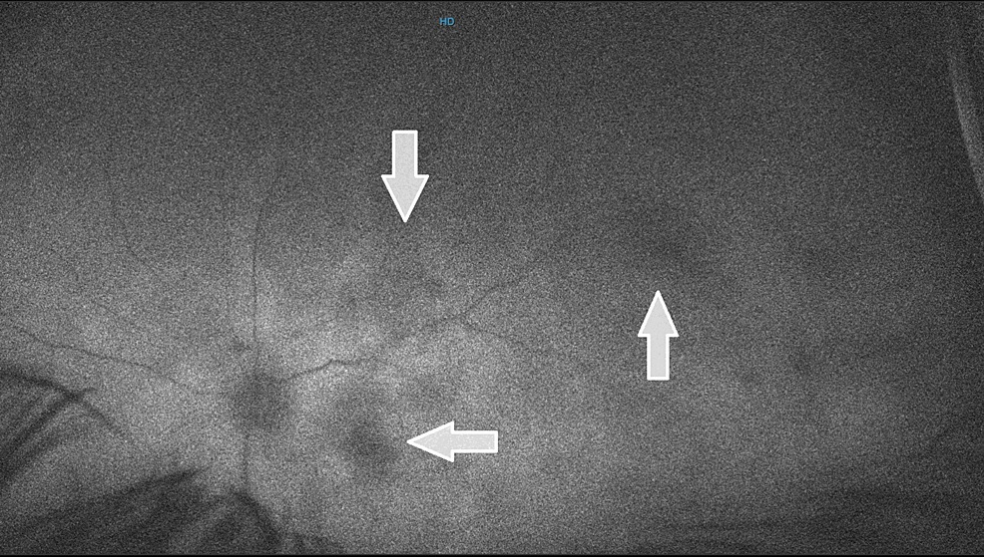
Fundus fluorescein angiography (FFA) showing ischemic areas (white arrows). HD, high-definition

**Figure 5:**
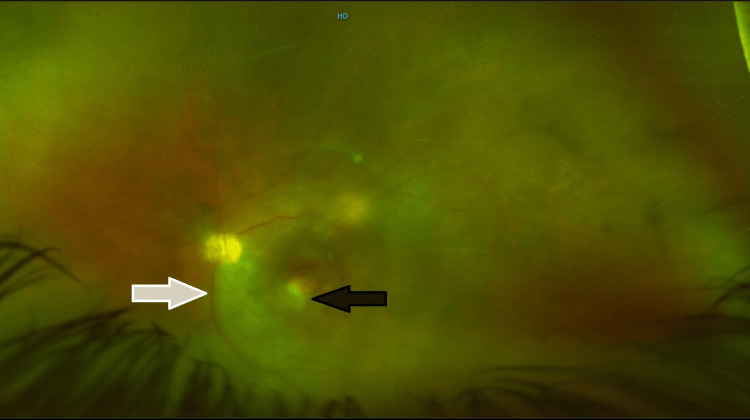
A color photograph of the fundus (left eye) showing macular involvement. Moreover, macular scarring (black arrow) and vasculitis (white arrow) can also be seen. HD, high-definition

**Figure 6:**
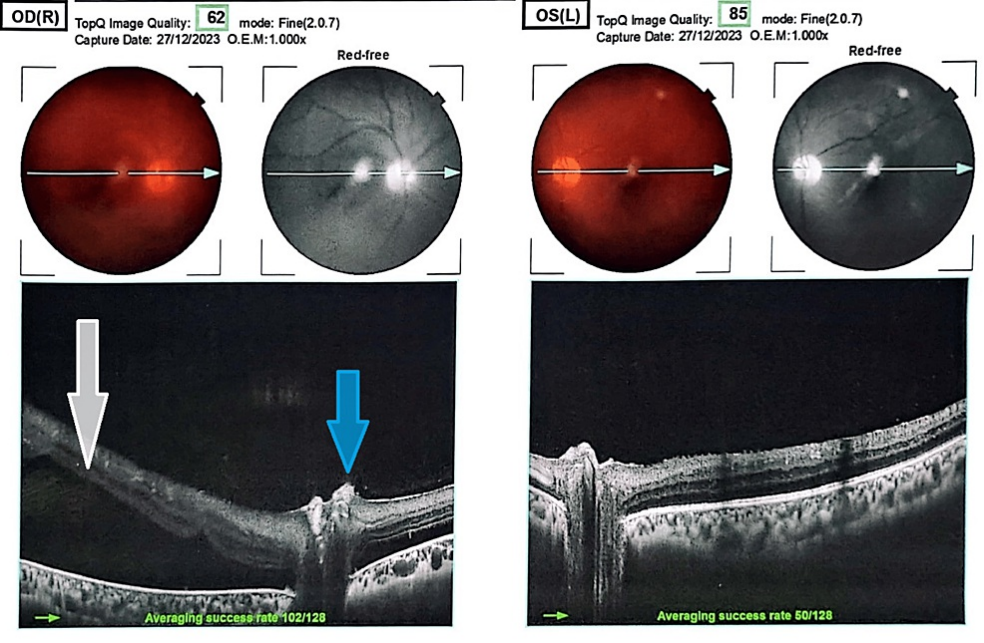
An optical coherence tomography (OCT) showing slight elevation of disc margin (blue arrow) with retinal detachment (white arrow) and epiretinal membrane on nasal side in the right eye. Left eye appears to be normal with loss of foveal contour. OD(R), oculus dexter (right eye); OEM, original equipment manufacturer; OS(L), oculus sinister (left eye)

Fundus examination of his left eye depicted massive ischemia of the retina on fundus fluorescein angiography (FFA) (Figure 4) and inflammation along with vasculitis. Ghost vessels with hazy fundus were seen inferiorly in the left eye, for which scatter laser was tried but to no avail. Vasculitis with retinitis in the left eye was also seen (Figure 5). Moreover, an optical coherence tomography (OCT) of his right eye revealed RD, whereas findings in the left eye were insignificant (Figure 6).

All the other investigations which included anti-nuclear antibody (ANA) test, antineutrophilic cytoplasmic autoantibody, cytoplasmic (c-ANCA) test, perinuclear anti-neutrophil cytoplasmic antibodies (p-ANCA) test, anti-human immunodeficiency virus (anti-HIV) test, venereal disease research laboratory (VDRL) test for syphilis, Mantoux test for tuberculosis, toxoplasma immunoglobulin-G (Ig-G) and immunoglobulin-E (Ig-E) tests, all came out to be negative and their values were well within the normal range.

## Discussion

BD is a rare, systemic disorder prevalence, which is seen in Mediterranean and Middle Eastern countries. BD is not frequently reported from the Indian subcontinent. It typically manifests itself predominantly in males, particularly during the third and fourth decades of their lives [8].

According to International Study Group criteria for BD [9], there should always be a recurrence of oral ulcers, which may present either as aphthous ulcers (minor or major) or as herpetiform ulcers. Additionally, two of the below-mentioned criteria must be fulfilled, including recurrent genital ulcers, ocular lesions such as uveitis, vitreous showing cells on examination, or vasculitis in the retina, dermal involvement like pseudofolliculitis, erythema nodosum, and papulopustular lesions, as well as a positive pathergy test.

For the sake of facilitating the early diagnosis of BD, an International Criteria for Behçet's disease (ICBD) has been proposed. ICBD consists of a particular scoring system based on the presenting signs and symptoms of the disease, with two points allocated for each: ocular lesions, genital ulcers, and oral ulcers. At the same time, one point each is assigned to lesions of skin, vessels, and nerves. According to ICBD, a score of more than or equal to four is highly suggestive of the diagnosis of BD [10].

Our patient had recurrent oral and genital ulceration and positive fundus examination findings, which included panuveitis, vasculitis, ischemic retina, and vitritis bilaterally, as well as inferior RD with FTMH in his right eye. The ICBD score was calculated for this particular case, which came out to be seven, thus strongly suggesting Behçet's disease.

Retinal vasculitis and chronic cystoid macular edema (CME) may be regarded as the potential risk factors in the development of MH and might explain the pathophysiology behind its development in BD.

In accordance with the criteria of International Study Group for diagnosing Behçet’s syndrome, it is mandatory for the patient to have recurrent ulcerations of oral cavity along with two of the other signs such as ulcerations of genitalia, lesions of the eye, lesions of the skin, or a positive pathergy test [11].

A notable improvement was seen in the patient’s uveitis in this case when treated with prednisolone, thus suggesting the efficacy of steroid therapy.

## Conclusions

Immunosuppressive therapy is the main course of treatment for Behçet’s disease. The earlier the diagnosis, the greater it will help in delaying the progression of the disease as well as preventing other complications. Although currently, no definitive treatment option is available for BD, the patients need to be counseled about the relapsing and remitting nature of the disease. The importance of regular follow-ups must be emphasized. Moreover, relevant specialties, such as dermatology, ophthalmology, internal medicine, neurology, and rheumatology, should collaborate with each other to help in improving the outcome of the patient due to the multi-systemic nature of BD.
